# The Mining Bulletin

**Published:** 1898-04

**Authors:** 


THE MINING BULLETIN is published by the Pennsylvania State College.

The March number of this interesting periodical has been received. Among other items of information we note that it has made an interesting exhibit of the coal production of Pennsylvania. Last year the production was 46,947,650 tons of anthracite and 54,672,500 tons of bituminous coal—sufficient in amount to fill three million freight cars, which in a train would be long enough to reach nearly twice around the globe.

Every 110,000 tons of anthracite coal mined and sold cost one life and more than two serious injuries, employing 226 miners. Every 360,000 tons of bituminous coal means the loss of one life and injuries to three others with the employment of nearly 600 men.

This journal is published for the benefit of the mining industry, and will be sent to any one desiring a copy.

